# Selective vulnerability of human-induced pluripotent stem cells to dihydroorotate dehydrogenase inhibition during mesenchymal stem/stromal cell purification

**DOI:** 10.3389/fcell.2023.1089945

**Published:** 2023-02-06

**Authors:** Ziadoon Al-Akashi, Denise Zujur, Daisuke Kamiya, Tomohisa Kato, Toru Kondo, Makoto Ikeya

**Affiliations:** ^1^ Center for iPS Cell Research and Application (CiRA), Kyoto University, Kyoto, Japan; ^2^ Takeda-CiRA Joint Program, Fujisawa, Kanagawa, Japan; ^3^ Medical Research Institute, Kanazawa Medical University, Kanazawa, Japan; ^4^ Institute for Genetic Medicine, Hokkaido University, Sapporo, Hokkaido, Japan

**Keywords:** mesenchymal stem/stromal cells (MSCs), induced mesenchymal stromal cells (iMSCs), induced pluripotent stem cells (hiPSC), pyrimidine biosynthesis, dihydroorotate dehydrogenase (DHODH), brequinar (BRQ), survival

## Abstract

The use of induced mesenchymal stem/stromal cells (iMSCs) derived from human induced pluripotent stem cells (hiPSCs) in regenerative medicine involves the risk of teratoma formation due to hiPSCs contamination in iMSCs. Therefore, eradicating the remaining undifferentiated hiPSCs is crucial for the effectiveness of the strategy. The present study demonstrates the Brequinar (BRQ)-induced inhibition of dihydroorotate dehydrogenase (DHODH), a key enzyme in *de novo* pyrimidine biosynthesis, selectively induces apoptosis, cell cycle arrest, and differentiation; furthermore, it promotes transcriptional changes and prevents the growth of 3-dimensional hiPSC aggregates. Contrastingly, BRQ-treated iMSCs showed no changes in survival, differentiation potential, or gene expression. The results suggest that BRQ is a potential agent for the effective purification of iMSCs from a mixed population of iMSCs and hiPSCs, which is a crucial step in successful iMSC-based therapy.

## Graphical Abstract

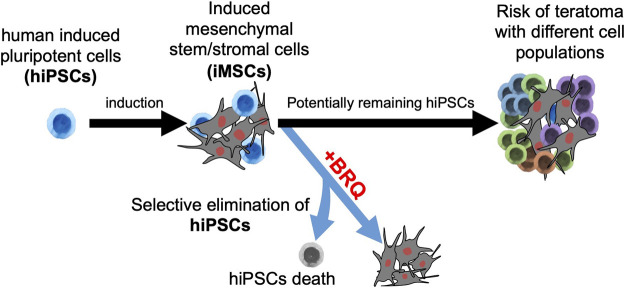

## 1 Introduction

Mesenchymal stem/stromal cells (MSCs) are present in almost all types of human tissues and contribute to tissue regeneration either directly or by paracrine-mediated effects (Han et al., 2019; Shammaa et al., 2020). MSC-based therapy is introduced in modern medicines. They are used for the treatment of graft-versus-host disease and are under investigation for their implementation in regulating the immune response in COVID-19 patients for the treatment of acute respiratory distress syndrome (ARDS), alleviating cardiac damage in post-myocardial infarction, and accelerating wound healing with increased vascularization (Wu et al., 2007; Duran et al., 2013; Liang et al., 2020).

Despite their advantages and wide spectrum of applications, there are limitations to MSC acquisition in terms of invasiveness, proliferation, and variation in biological characteristics (Abbas et al., 2019; Kozlowska et al., 2019; Sarkar et al., 2019). Induced MSCs (iMSCs) derived from human induced pluripotent stem cells (hiPSCs) provide rejuvenated, easily scalable, and homogeneous populations of MSCs (Eto et al., 2018; Spitzhorn et al., 2019; Zhang et al., 2021). However, hiPSC-derived cell therapy carries the risk of teratoma formation if some pluripotent cells remain undifferentiated (Ben-David and Benvenisty, 2011). Therefore, the complete eradication of unwanted hiPSCs is a crucial step in safe iMSC-based therapies.

Different methods have been proposed for the elimination of pluripotent cells by inhibiting fatty acid synthesis, genetic modulation, changing the culture medium concentration of certain amino acids, or inhibiting the rate-limiting enzyme in *de novo* pyrimidine synthesis (Nagashima et al., 2018; Kimura et al., 2019; Tanosaki et al., 2020; Kondo, 2021). Fast proliferating cells rely solely on the *de novo* pyrimidine biosynthesis pathway to meet the high demand for nitrogenous bases (Walter and Herr, 2022). Dihydroorotate dehydrogenase (DHODH) is a rate-limiting enzyme in the *de novo* pyrimidine biosynthesis pathway; thus, depletion of cellular pyrimidines is achieved by inhibition of DHODH (Desler et al., 2010; Chen et al., 2019). The expression of DHODH is regulated by the oncogene MYC, which is significantly upregulated in cancer cells and pluripotent cells. DHODH inhibitors suppress MYC expression, presumably due to the induction of apoptosis and cell cycle arrest in cancer cells (Dorasamy et al., 2017; Christian et al., 2019).

Although it was previously reported that DHODH inhibitors selectively eliminated murine pluripotent cells, it is still unknown and worth confirming in human cells. We hypothesized that *de novo* pyrimidine biosynthesis is indispensable for the high nitrogenous base demand in hiPSCs and that iMSCs can utilize the pyrimidine salvage pathway. In this study, we treated iMSCs with the potent DHODH inhibitor Brequinar (BRQ) and analyzed the cell survival, differentiation potential, gene expression, and functional characteristics to determine the effectivity and safety of using the DHODH inhibitor for the elimination of unwanted hiPSCs, which will provide a fundamental step for the future of iMSC-base therapy.

## 2 Materials and methods

### 2.1 iPSCs culture

For the present study, hiPSCs (1231A3) reprogrammed by episomal vectors (Nakagawa et al., 2014), RPCiPS771-2 reprogrammed by RNA (Stemgent), Ff-XT28s05-Abo_To reprogrammed by episomal vectors and knockout of HLA-A,B, and CIITA genes (provided by CiRA Foundation) were cultured in plastic dishes coated with iMatrix-511 silk (Nippi, 892021) fed with StemFit AK03N (Ajinomoto) under xeno-free culture conditions. Cells were dissociated into a cell suspension using Accutase^®^ (Sigma Aldrich, A6964) and passaged when they reached more than 70% confluence.

### 2.2 iPSC cell aggregates assay

For 3-dimensional cell aggregates experiment, hiPSCs were dissociated into cell suspensions and seeded into 96 well U-bottom ultra-low attachment plates (PrimeSurface^®^, Sumitomo Bakelite, MS-9096U) in StemFit Basic03 (equivalent to AK03N without bFGF, Ajinomoto) in the presence of ROCK inhibitor (Y-27632, Wako, 034-24024). After aggregates with clear edges were formed, the culture medium was replaced with DMEM/F12 supplemented with 0.1% human serum albumin (HSA, Wako, 014-21543) and 10 ng bFGF (R&D, 236-EG). Aggregate cross-sectional area sizes were measured using Fiji ImageJ software (Schindelin et al., 2012). 1231A3 hiPSCs were used in all experiments unless otherwise stated.

### 2.3 iMSCs culture

iMSCs were generated by inducing hiPSCs into neural crest cells (NCCs), and then into iMSCs (Kamiya et al., 2022), which were then maintained in a plastic dish coated with Fibronectin (Chemicon, FC010-10 MG) and fed with PRIME-XV^®^MSC Expansion XSFM (IrvineScientific, 91149). Analyses were performed during passages 3—5.

### 2.4 iMSCs differentiation

For the adipogenic differentiation of iMSCs (Figure 5D), 7 days BRQ (Selleck, S3565)-or DMSO (Nacalai,13408-64)-pretreated iMSCs were seeded on fibronectin-coated wells, and cells were maintained until they reached full confluence, then adipogenic induction medium made of DMEM (High Glucose, Nacalai, 08459-64), 10% fetal bovine serum (FBS) (Nichirei,171012), 0.5% penicillin/streptomycin (GIBCO, 15140122), 10 μg/ml insulin (Wako, 097-06474), 1 µM dexamethasone (Wako, 047-18863), 200 µM indomethacin (Wako, 093-02473), and 500 µM IBMX (Wako, 095-03413) were added to it. After 3 weeks of adipogenic induction, cells were stained with Oil Red O (Nacalai, 25633-92). For osteogenic differentiation (Figure 5E), 7 days BRQ-or DMSO-pretreated iMSCs were seeded on gelatin-coated wells, maintained until they reached full confluence, and then treated with osteogenic induction medium made of MEM-Alpha GlutaMAX (GIBCO, 32571-036), 10% FBS, 0.5% penicillin/streptomycin, β-glycerophosphate disodium salt hydrate (SIGMA, G9422), and 100 nM dexamethasone. After 3 weeks of osteogenic induction, the cells were stained with Alizarin Red S solution (Muto Pure Chemicals, 17971). For chondrogenic differentiation using the micromass method (Figure 5F), a drop of 5 µl of 7 days BRQ- or DMSO-pretreated iMSCs suspension in the center of a well (24-well plate) coated with fibronectin. After incubation at 37°C for 1 h, the hMSC Chondrogenic Differentiation BulletKit (Lonza, PT-3003), 100 ng/ml TGFB3 (R&D, 243-B3-200), and 100 ng/ml BMP7 (R&D, 354-BP-010V) were added. After 7 days of differentiation, Alcian blue solution (pH = 1; Muto Pure Chemicals, 40862) was added. We used DMSO as the control treatment at similar volume to BRQ since BRQ was dissolved in DMSO.

### 2.5 Cell survival

To examine cell survival, cells were cultured with the DHODH inhibitors teriflunomide (Selleck, S4169), ASLAN003 (Selleck, S9721-5 MG), and BRQ. For the rescue experiment, 100 µM of uridine (Sigma Aldrich, U3003-5G) was added. Then, using a highly water-soluble tetrazolium salt-based cell counting kit (CCK-8 Dojindo, CK04-05), absorption was detected using a 450 nm plate reader (PerkinElmer, 2104 EnVision). The survival percentage was calculated relative to the non-treated control and by subtracting the background absorbance value.

### 2.6 Flow cytometry analysis

Apoptosis was evaluated using the Annexin V-FITC Apoptosis Detection Kit (Nacalai, 15342-54), which includes an Annexin V-FITC conjugate and propidium iodide (PI) according to the protocol provided by the manufacturer, and was further analyzed using flow cytometry. For the cell cycle analysis, Cell Cycle Assay Solution Deep Red (Dojindo, C548) was used; the solution provided with the kit was added to the cell suspension, followed by incubation according to the manufacturer’s protocol, and a flow cytometric analysis. For staining, CD105-APC (eBioscience, 17-1057-42), CD90-PE (eBioscience, 555596), CD73-PE (eBioscience, 550257), CD44-PE (eBioscience, 550989), CD34-PE (eBioscience, 560941), CD31-Alexa Fluor^®^ 647 (BioLegend, 303112), and TRA-1-60-PE (Thermo Fisher, 12-8863-82) (1:50) was added to the cell suspension and incubated for 30 min before the flow cytometric analysis. BD FACSAria™ III was used to detect fluorescence. Results were plotted using FlowJo_v10.8.1 software.

### 2.7 Immunochemistry

Cells were fixed using 4% PFA, stained with anti-POU5F1 (Santa Cruz Biotechnology, sc-5279), and detected using Alexa Fluor 555 goat anti-mouse (Thermo Fisher, A21422) and DAPI counterstaining. They were visualized and imaged with a Keyence microscope system (BZ-X710 and BZ-X810).

### 2.8 Real-time quantitative PCR analysis

RNA samples were extracted using the RNeasy^®^ Mini kit (Qiagen) and reverse-transcribed to cDNA. Real-time quantitative PCR (RT-qPCR) was performed using THUNDERBIRD™ Next SYBR^®^ (TOYOBO, QPX-201), QuantStudio™ 3 Real-Time PCR System, and QuantStudio™ 7 Flex Real-Time PCR System (Applied Biosystems) with specifically designed primers (Supplementary Table S1). Data from three biological replicates were analyzed to calculate the relative fold change (2^−ΔΔCT^). GraphPad Prism 9 software was used to plot the graphs.

### 2.9 RNAseq data analysis

Libraries were prepared using the Ion AmpliSeq Transcriptome Human Gene Expression Panel, Chef-Ready Kit (A31446), and Ion Torrent Ion Chef Instrument (4484177) (Thermo Fisher Scientific, Tokyo, Japan). In brief, 10 ng of RNA was reverse transcribed with SuperScript VILO cDNA Synthesis Kit (11754050), PCR conditions were 13 cycles of 16 min extension reaction using 10 ng of cDNA, qPCR Ion Library TaqMan Quantitation Kit (4468802). 50 p.m. was used for template preparation and sequencing using Ion Chef. Sequencing was performed using the Ion Torrent Ion GeneStudio S5 Prime System. Then, the count data analyses were performed using R studio with the ‘DESeq2’ package normalization method for the detection of significantly (*p* ≤ 0.05) differentially expressed genes (Love et al., 2014).

### 2.10 Statistical analysis

For statistical analyses, ANOVA with Dunnett’s multiple comparisons test or unpaired parametric Welch’s *t*-test was performed using GraphPad Prism 9 software (*p ≤ 0.05, **p ≤ 0.01, ***p ≤ 0.001, and ****p ≤ 0.0001).

## 3 Results

### 3.1 Vulnerability of hiPSCs to DHODH inhibition

**FIGURE 1:**
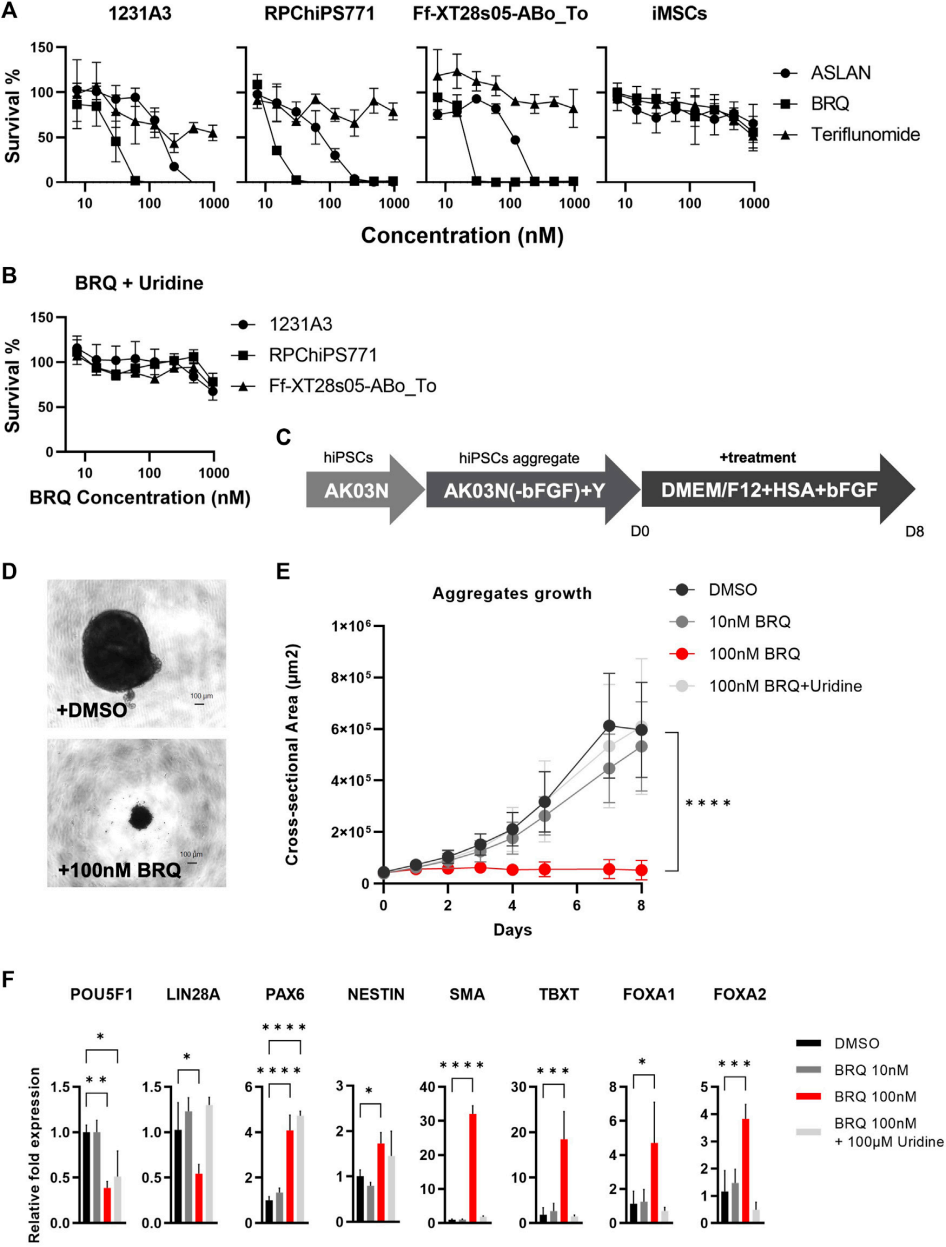
Vulnerability of hiPSCs to DHODH inhibition **(A)** Survival measurement using water-soluble formazan dye after the treatment with DHODH inhibitors, Teriflunomide (Triangle), ASLAN (Circle), and Brequinar (Square) for 7 days on 1231A3, RPChiPS771, Ff-XT28s05-ABo_To hiPSCs lines and iMSCs (From left to right). **(B)** Rescued survival of 1231A3 (Circle), RPChiPS771 (Square), and Ff-XT28s05-ABo_To (Triangle) BRQ-treated hiPSCs lines by 100 µM Uridine supplementation. Data plotted as mean with error bar S. D, minimum of triplicates per hiPSCs line and triplicates of three biological repeats of the iMSCs. **(C)** Schematic representation of iPS cell aggregates formation, hiPSCs were collected in single cell suspension then cultured in ultra-low attachment U-bottom 96-well plates in StemFit Basic03 in the presence of ROCK inhibitor (Y) for 2–3 days until aggregates with clear edges are formed. Medium then was replaced with DMEM/F-12 supplemented with 0.1% HSA and 10 ng bFGF and **(D)** phase contrast images were taken, then the **(E)** area of each aggregate was measured, dots represent mean and error bar is S. D, n = 6, biological replicates. **(F)** Relative mRNA expression of pluripotency markers (POU5F1 and LIN28), ectodermal markers (PAX6 and NESTIN), mesodermal markers (SMA and TBXT), and endodermal markers (FOXA1 and FOXA2), mRNA of minimum 4 aggregates were extracted per sample after 3 days of treatment then expression was detected by RT-qPCR, DMSO (Black), 10 nM BRQ (Dark gray), 100 nM BRQ (Red) and 100 nM BRQ with 100 µM Uridine (Light gray). Bar: mean, Error bar: S. D, n = 3, biological replicates.

To test the viability of hiPSCs treated with different DHODH inhibitors, we cultured cells in 96-well plates and treated cells in each experiment with one of the different DHODH inhibitors, teriflunomide, ASLAN003, and BRQ, for 7 days at different concentrations. After 7 days of treatment, the CCK-8 kit solution was added and incubated for 4 h. Absorbance was measured using a 450 nm filter plate reader. Approximately 50 nM of BRQ resulted in the complete eradication of hiPSCs, which was more potent than ASLAN (250–500 nM), while the hiPSCs showed higher tolerance to teriflunomide and 50% survival even at the highest concentration tested (Figure 1A). Contrastingly, iMSCs were more resilient to the DHODH inhibitors and showed >50% survival, even at the highest concentration tested. hiPSCs were rescued by adding 100 µM uridine, which suppressed the inhibitory effects of BRQ (Figure 1B). The effects of DHODH inhibitors and the uridine-induced rescue from the BRQ effect were similar in the three hiPSC lines tested. These results indicate that 100 nM BRQ is sufficient to fully eradicate hiPSCs, but not iMSCs. While the effect of BRQ on hiPSCs can be prevented by adding uridine that saturates the salvage pathways, iMSCs utilize the salvage pathway for pyrimidine biosynthesis, which is sufficient to sustain the viability of iMSCs without uridine supplementation.

To reveal the effects of BRQ on 3-dimensional (3D) cell condition, we cultured 1231A3 hiPSCs in 96-well U-bottom plates as 3D cell aggregates, and then replaced the culture medium with DMEM/F12 medium supplemented with 0.1% HSA and 10 ng bFGF to allow the hiPSCs to spontaneously differentiate. Subsequently, the treatment of cell aggregates with 100 nM BRQ, 10 nM BRQ, 100 nM BRQ supplemented with 100 µM uridine, and DMSO control was done for 8 days (Figure 1C). Aggregates treated with 100 nM BRQ failed to grow and were significantly smaller (*p* < 0.0001) than DMSO-treated aggregates (Figures 1D, E). We collected samples at an early stage of treatment (day 3) and extracted mRNA for gene expression analyses. Despite their smaller sizes, BRQ-treated aggregates revealed significantly upregulated ectodermal (PAX6 and NESTIN, *p* < 0.0001 and *p* = 0.0499), mesodermal (SMA and TBXT, *p* < 0.0001 and *p* = 0.0007), and endodermal (FOXA1 and FOXA2, *p* = 0.0249 and *p* = 0.0009) gene markers of the germ layers and significantly downregulated of the pluripotency marker (POU5F1, *p* = 0.0044). The 100 nM BRQ-treated aggregates also revealed significant downregulation of LIN28A (*p* = 0.0268) which is a marker for residual undifferentiated hiPSCs (Kuroda et al., 2012) (Figure 1F). These results suggest that BRQ is effective in eliminating undifferentiated hiPSCs, preventing the growth of hiPSC aggregates and spare differentiated cells.

### 3.2 BRQ selectively eliminates hiPSCs in iMSC-iPSC mixed culture

**FIGURE 2:**
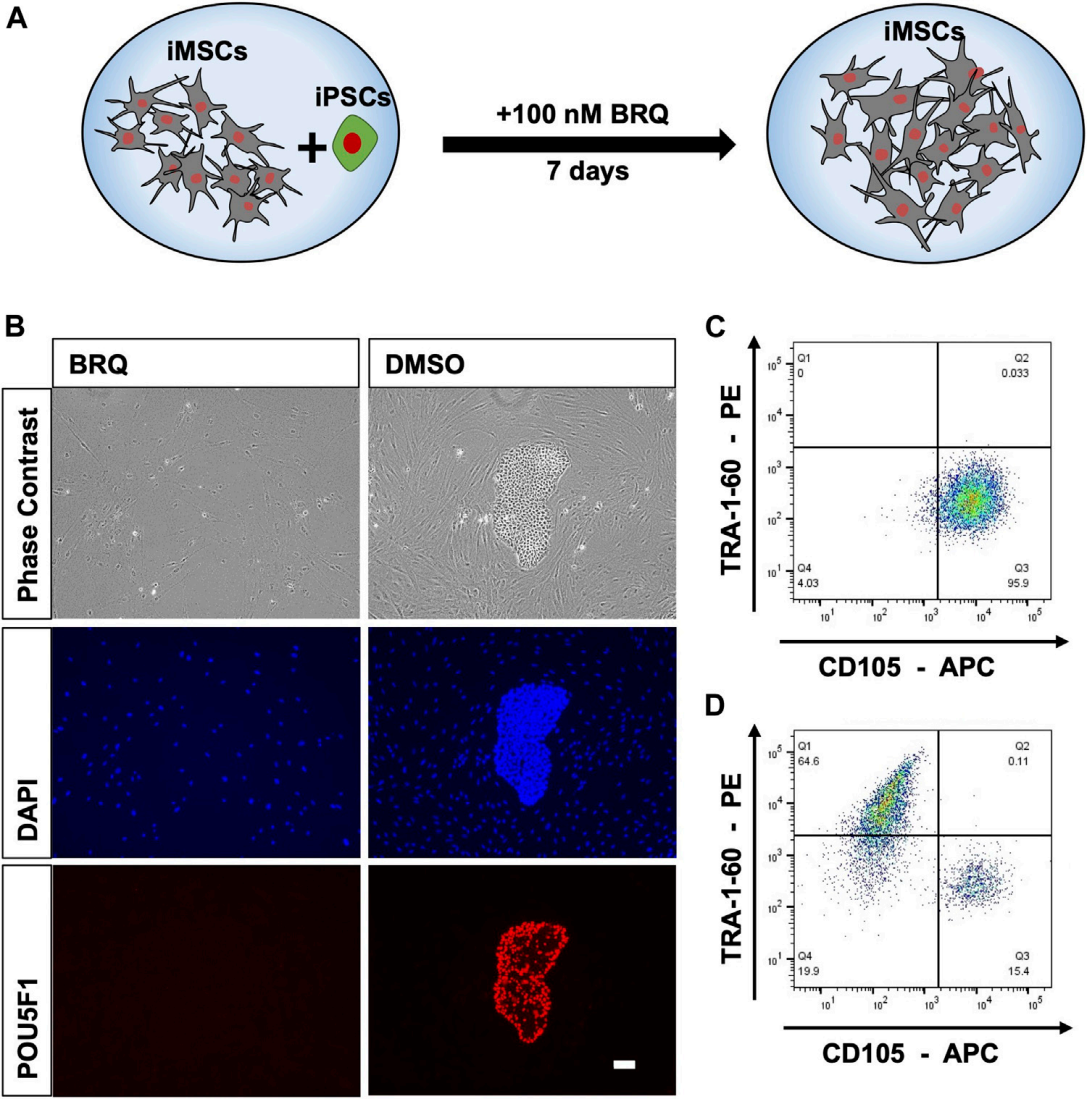
Selective elimination of hiPSCs by BRQ in iMSC-iPSC mixed culture. **(A)** Schematic representation of the mixed culture condition by mixing iMSCs with 10% hiPSCs and culturing both cell types in the presence of BRQ for 7 days. **(B)** iMSCs mixed with 10% hiPSCs cultured for 1 week in the presence of 100 nM BRQ or DMSO control. Cells were fixated using 4% PFA then immunostained using anti-POU5F1(Red) antibody and nuclear stain DAPI (Blue), scale bar 100 µm. Mixed culture was repeated, **(C)** BRQ- and **(D)** DMSO-treated cells were stained with surface antibodies CD105 and TRA-1-60. Cells were then passed through the cell sorter for analysis. Experiments were performed in three biological repeats, representative figures.

To further investigate the effectiveness of BRQ in application settings and cell type selectivity, we intentionally contaminated iMSCs derived from the 1231A3 hiPSC line with 10% hiPSCs from the same cell line, and co-cultured both cell types for a week in one culture well in the presence of BRQ or DMSO (Figure 2A). The mixed cells were cultured in 6-well plates coated with both fibronectin and iMatrix-511, fed with Stemfit Basic03, and then treated with 100 nM BRQ or DMSO. We visually examined cell cultures and found that 100 nM BRQ-treated mixed culture prevented hiPSCs from proliferating and forming colonies during the 7 days of cell culture, whereas colonies were formed in DMSO-treated mixed culture (Figure 2B). The cells were fixed with 4% PFA, immunostained using anti-POU5F1 antibody and nuclear stain (DAPI), and fluorescence microscopic imaging was done. The results showed that the BRQ-treated mixed culture fully eliminated hiPSCs, as indicated by the absence of POU5F1 positive colonies. Similar results were obtained using cell sorting analyses. The colonies were dissociated to form a single-cell suspension and stained with TRA-1-60 and CD105 conjugated antibodies, which are surface markers for human iPSCs and iMSCs, respectively. The stained cells were then passed through a cell sorter for analysis. The results revealed the absence of TRA-1-60 positive cells in BRQ-treated samples (Figure 2C), and the presence of 64.6% of positive cells in the DMSO control (Figure 2D) was detected. These results show the effectiveness of BRQ in eliminating unwanted hiPSCs, even in the presence of different cells in the same culture well, by selectively eliminating hiPSCs and sparing iMSCs derived from the same 1231A3 hiPSC line.

### 3.3 BRQ-treated hiPSCs show relevant transcriptional changes

**FIGURE 3:**
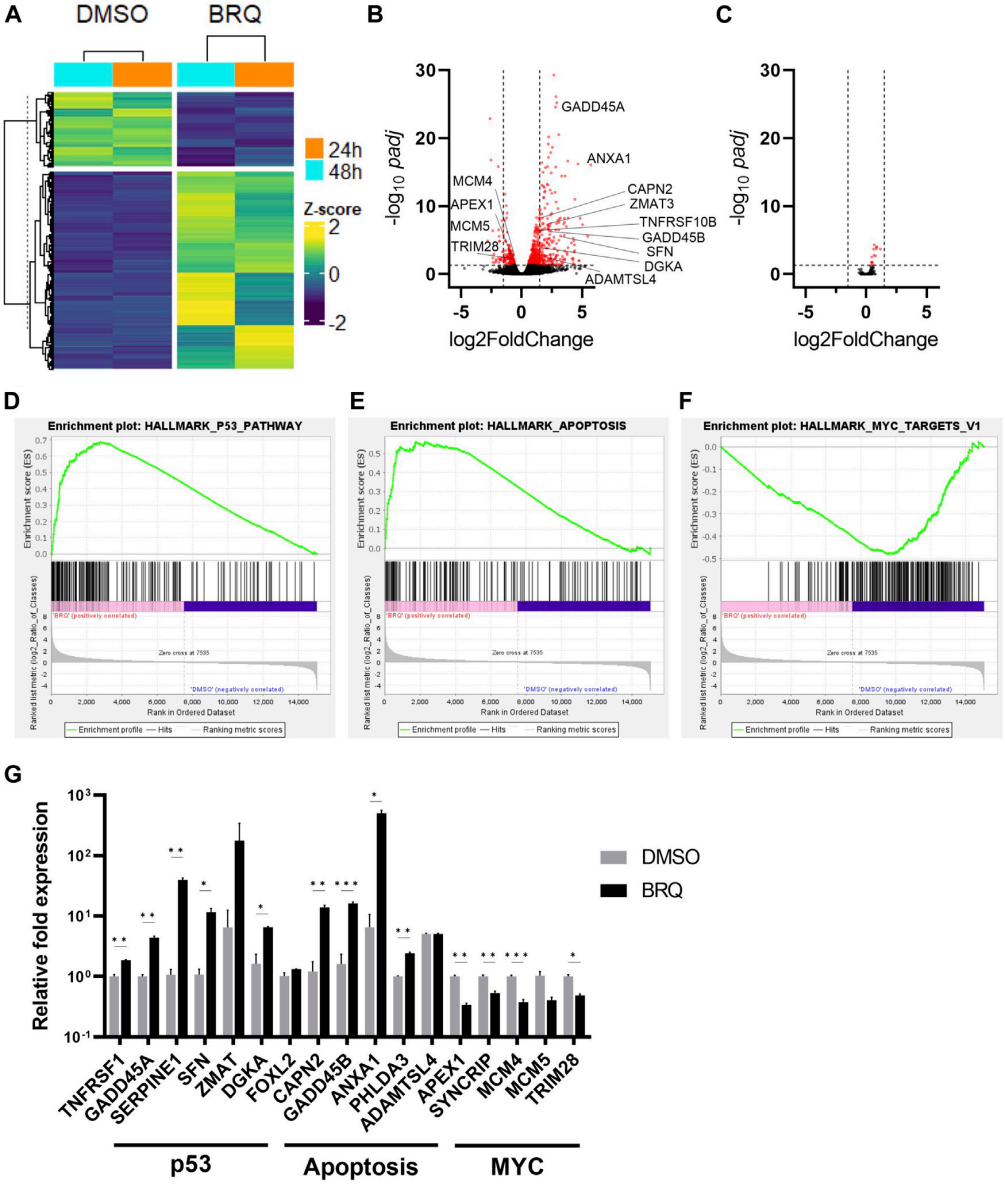
Relevant transcriptional changes of BRQ-treated hiPSCs. **(A)** hiPSCs treated for 24 h and 48 h, hieratical clustering of samples of the significantly differentially expressed genes (100 nM BRQ vs. DMSO control), bright yellow color indicates higher expression of gene and blue for lower expression. Volcano plots of significance (-log10 *padj*) vs. log2 fold change showing the differentially expressed genes of 100 nM BRQ vs. DMSO control of **(B)** hiPSCs, and **(C)** iMSCs. Red color dots indicate genes showing significantly differentially expressed genes (*p* ≤ 0.05 horizontal dashed) that are transcriptionally downregulated or upregulated (log2Fold change ≥1.5 or ≤ -1.5 vertical dashed lines). Gene Set Enrichment Analysis (GSEA) of the normalized counts data of BRQ- and DMSO-treated hiPSCs shows a correlation with **(D)** Apoptosis targets, **(E)** p53 pathway, and **(F)** MYC targets. **(G)** 48 h BRQ-treated hiPSCs relative mRNA expression of Apoptosis, p53, and MYC targets detected by RT-qPCR, bars indicate the mean, error bars indicate S.D., n = 3, biological replicates.

To reveal the effect of BRQ on global gene expression, we performed next-generation RNA sequencing (RNAseq) of hiPSCs treated with 100 nM BRQ for 24 h and 48 h, and the normalized count data of the differentially expressed genes (DEGs) were analyzed. The BRQ and DMSO samples revealed distinct clusters of highly and less expressed genes, and both samples of each condition showed two hierarchical clusters (Figure 3A). Gene expression analysis of BRQ vs. DMSO samples revealed 646 significant (*p* ≤ 0.05) DEGs. Approximately 33.7% of the genes were transcriptionally upregulated (log_2_ fold-change ≥1.5), and approximately 4.33% of genes were downregulated (log_2_ fold-change ≤ -1.5) in BRQ-treated samples. Results were plotted to show the significance vs. log fold change (Figure 3B), and we found that the genes relevant to DNA damage and cell cycle regulation, regulation of apoptosis, and MYC targets were significantly differentially expressed (Fornace et al., 1989; Zeller et al., 2003; Yang et al., 2006; Kawase et al., 2009; Weyd et al., 2013; Dubail and Apte, 2015; Yang et al., 2020). Contrastingly, RNAseq data analyses of BRQ-treated iMSCs revealed only 14 DEGs (Supplementary Figure S1A), and expressions of none of these genes were altered at the transcriptional level (log_2_ fold-change ≥1.5 or ≤ -1.5) (Figure 3C).

Gene Set Enrichment Analysis (GSEA) using the hallmark data set against the normalized count data of BRQ- and DMSO-treated hiPSCs revealed a positive correlation of apoptosis and p53, and a negative correlation of MYC targets with BRQ-treated samples’ expression data (Figures 3C–E). Moreover, RT-qPCR analyses revealed upregulation of genes relevant to p53 and apoptosis pathways, and downregulation of MYC targets in 48 h BRQ-treated hiPSCs relative to DMSO control (Figure 3G), however, 7 days BRQ-treated iMSCs showed relatively unchanged expression (Supplementary Figure S1B), which supported the findings in RNAseq data. These results suggest that the BRQ treated hiPSCs lost their oncogenic property and under the instructions of programmed cell death (PCD).

### 3.4 BRQ induces apoptosis and cell cycle arrest in hiPSCs but not in iMSCs

**FIGURE 4:**
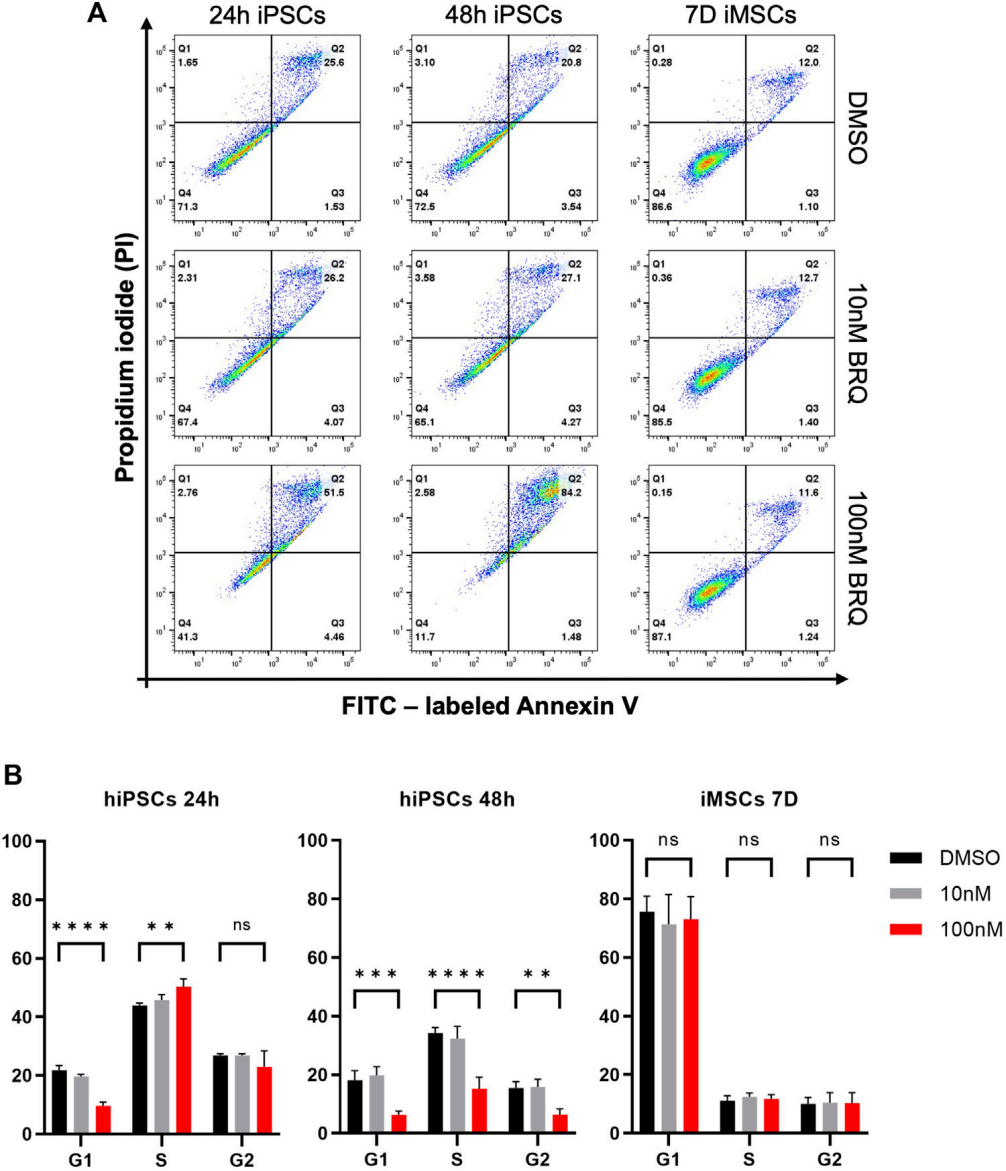
Induction of apoptosis and cell cycle arrest in hiPSCs but not in iMSCs by BRQ treatment. hiPSCs were cultured until 70% confluence was reached, and iMSCs were treated directly after plating for 7 days. **(A)** Apoptosis detection in 24 h and 48 h BRQ- and DMSO-treated hiPSCs and 7 days BRQ and DMSO-treated iMSCs. Dissociated cells were collected in cell suspension, stained with FITC labeled Annexin V and propidium iodide (PI) using an Annexin Apoptosis Detection Kit, and analyzed by flow cytometry. n = 3, representative figures. **(B)** Cell cycle analysis of 24 h and 48 h BRQ- and DMSO-treated hiPSCs, and 7 days BRQ- and DMSO-treated iMSCs using a Cell Cycle Assay kit and analyzed using flow cytometry. n = 3, biological replicates.

To further investigate the cause of death in BRQ-treated cells, we tested the 24 h and 48 h BRQ-treated hiPSCs and 7 days BRQ-treated iMSCs by adding Annexin-V and propidium iodide (PI). Damage to membrane lipids permits the Annexin-V binding, whereas fully damaged lipid membranes expose the cells, allowing PI binding to the cell content, indicating that the cells underwent PCD. If only PI was detected, the cells were considered to have died from necrosis. It is important to note that hiPSCs have higher apoptosis than iMSCs in control conditions. hiPSCs in small colonies or when cells are dissociated are prone to be eliminated by apoptosis due to mechanical stress. On the other hand, stress induced proliferation and did not affect apoptosis in the MSCs (Choi et al., 2007; Ohgushi et al., 2010; Frank et al., 2016). The results demonstrated a shift of hiPSCs treated with 100 nM BRQ to the double positive quadrant (51.2% at 24 h and 84.2% at 48 h), in which cells were positive for both Annexin-V and PI, indicating the late-apoptotic stage, while the iMSCs treated for 7 days revealed no marked change in the percentages of living cells or cells undergoing apoptosis (Figure 4A). Cell cycle analysis revealed that BRQ-treated hiPSCs caused cell cycle arrest in the S/G2 phase after 48 h of treatment, with a significant decrease in the percentage of cells in the G1 phase, while iMSCs treatment for 7 days showed no changes in the cell cycle (Figure 4B). These data further verify that BRQ treatment causes cell cycle arrest and commences PCD only in hiPSCs and the resilience of iMSCs to BRQ effects.

### 3.5 BRQ treated iMSCs maintain their characteristics and differentiation potentials

**FIGURE 5:**
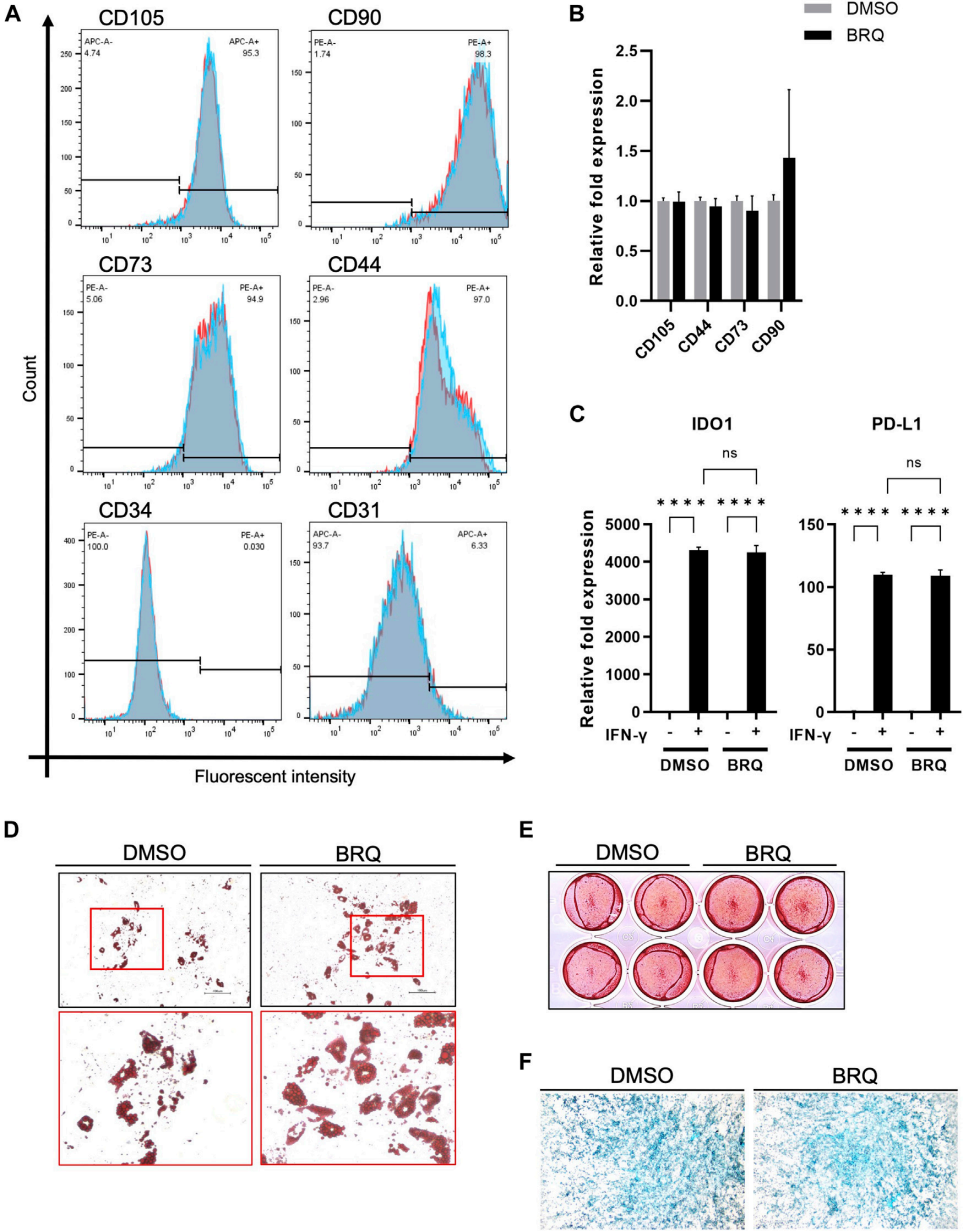
BRQ-treated iMSCs maintain their characteristics and differentiation potentials **(A)** Overlay histogram of the CD105, CD90, CD73, CD44, CD34, and CD31 in DMSO (Blue) and BRQ (Red) treated iMSCs for 7 days, detected by staining with conjugated antibodies then passed through cell sorter for analysis. n = 3, biological replicates, representative figures. **(B)** mRNA expression of CD105, CD90, CD73 and CD44 detected by RT-qPCR, 100 nM BRQ 7 days treated iMSCs (Black) relative fold expression to DMSO treated iMSCs (Gray). **(C)** mRNA expression of IDO1 (left) and PD-L1 (right) in DMSO pretreated iMSC or BRQ pretreated iMSCs with or without 10 ng/ml IFN-γ for 24 h mRNA expression relative to DMSO without IFN-γ of each gene was analyzed using RT-qPCR. Bars indicate the mean, error bar is SD, n = 3, biological replicates. Differentiation of BRQ and DMSO pretreated iMSCs into **(D)** adipocytes detected by Oil red staining, **(E)**, osteoblasts detected by Alizarin red staining, and **(F)** magnified image of the chondrocytes micromass detected by Alcian Blue staining.

We investigated whether BRQ-treated iMSCs maintained their gene expression profiles. For 7 days, iMSCs were treated with 100 nM BRQ or DMSO. On the day 7, dissociated cell suspensions were collected and stained with MSC-specific positive surface markers CD105, CD90, CD73, and CD44 in addition to the MSC-specific negative surface markers CD34, and CD31; stained cells passed through a cell sorter were analyzed. iMSCs treated with BRQ expressed a fluorescence pattern similar to that of the DMSO control for the positive and the negative markers (Figure 5A). Although CD31 is a negative marker for iMSCs, we detected a slight percentage (6.33%) of cells that were positive for CD31. CD31 is expressed on the surface of endothelial cells and platelets (Duncan et al., 1999). Therefore, low detection level of CD31^+^ cells in iMSCs suggested partial differentiation of iMSCs into endothelial cells, and may be due to the fact that the iMSCs were derived from NCCs, one of whose derivatives is endothelial cells, or that the parent hiPSCs from which the iMSCs were derived were induced from a sample of peripheral blood. To further confirm these similarities, we evaluated mRNA expression of the same MSCs markers; BRQ-treated iMSCs showed relatively similar gene expression to that of the control (Figure 5B). Previous studies revealed that MSCs treated with interferon-γ (IFN-γ) showed upregulation of immunomodulatory genes IDO1 and PD-L1 granting MSCs their potential application in inflammatory diseases (Duncan et al., 1999; Wobma et al., 2018).Therefore, we supplemented the 100 nM BRQ pretreated iMSCs with IFN-γ then checked their mRNA expression (Figure 5C). The results showed that BRQ pretreated iMSCs maintained their reactivity to IFN-γ significantly (*p* < 0.0001), upregulating both IDO1 and PD-L1. The induced expression was similar to that observed in the DMSO control. Furthermore, iMSCs are multipotent cells that can differentiate into linage-specific cells, such as chondrocytes, osteoblasts, and adipocytes (Jones and McTaggart, 2008). We examined the differentiation potential of iMSCs after 7 days of BRQ treatment (Figures 5D–F), and these cells were detected to maintain their differentiation potential and were successfully differentiated into the linage-specific cells of adipocytes, osteoblasts, and chondrocytes, as confirmed by Alcian blue, Alizarin red, and Oil red O staining analyses, respectively.

## 4 Discussion

The stepwise induction of iMSCs from hiPSCs is controlled through selective cell sorting which utilizes specific antibody-mediated selection to eliminate unwanted cell populations (Kamiya et al., 2022). However, hiPSCs potentially remain undifferentiated and are able to evade selective cell sorting, potentially giving rise to teratomas, especially if large numbers of cells are transplanted in clinical settings (Ben-David and Benvenisty, 2011). Our study revealed the successful selective elimination of hiPSCs by BRQ-induced inhibition of the enzyme DHODH during *de novo* pyrimidine synthesis.

Here, we demonstrated that BRQ-induced inhibition of DHODH in hiPSCs prevented cell survival, caused cell cycle arrest, and induced apoptosis. Although *in vivo* teratoma formation assay is still the golden standard for the assessment of iPSCs pluripotency (Dominici et al., 2006). 100 nM BRQ eliminated all the living cells after few days, and it was not possible to obtain the required number of cells for the assay. Therefore, we allowed the hiPSCs to spontaneously differentiate as 3D aggregates utilizing the features of iPSCs to differentiate into cells of the three germ layers (ectoderm, mesoderm, and endoderm), and exploited suboptimal spontaneous differentiation in which some cells retained pluripotency (Hentze et al., 2009). It was previously reported that iPSCs cultured in mouse metanephron as a scaffold allowed the stem cells to differentiate without immune rejection and formed *in vitro* teratoma assay (Yamamoto et al., 2018). Similarly in our condition we applied ultra-low attachment plastic plates that sustained the cells in suspension forming aggregates of differentiated and undifferentiated iPSCs which revealed that treatment with 100 nM BRQ completely prevented growth of those aggregates. Remarkably, the 100 nM BRQ-treated aggregates, despite their smaller sizes, also showed higher gene expression of germ layer markers than that in control. This could potentially mean that the remaining cells in the 100 nM BRQ-treated aggregates had enhanced differentiation potential which could be the result of BRQ-induced differentiation due to cellular stress with the BRQ-sparing only differentiated cells and allowing those cells to proliferate. Interestingly, the BRQ-treated, Uridine-rescue aggregates revealed distinct upregulation of ectodermal marker. Furthermore, next-generation RNA sequencing indicated positive enrichment of apoptosis and p53 pathways in BRQ-treated hiPSCs, while negative enrichment of the MYC target pathway was detected. These findings demonstrate that the effect of pyrimidine bases depletion caused by BRQ drove the cells into arrest and initiation of programmed cell death with loss of tumorgenicity/pluripotency. Our findings were parallel with previous reports on the effects of BRQ on cancer stem cells and mouse pluripotent stem cells. Contrastingly, iMSCs were resilient to the effects of BRQ. After 1 week of treatment with 100 nM BRQ, iMSCs maintained their gene expression profile, lineage-specific differentiation potential, and IFN-γ-induced expression of IDO1 and PD-L1. RNA sequencing data revealed only 14 significantly differentially expressed genes, none of which showed a fold change greater than 1.5. This novel finding indicates that the pyrimidine requirement of iMSCs for proliferation is sufficed only by the salvage pathway, therefore inhibited key enzyme for the *de novo* pyrimidine synthesis (DHODH) sustained the status of the iMSC; so far, this criterion of iMSCs was not revealed by previous studies.

Cells acquire pyrimidine bases *via* either *de novo* biosynthesis or the salvage pathway. Highly proliferative cells (such as cancer or pluripotent cells) share a similar preference depending on the *de novo* pathway for pyrimidine biosynthesis, which is regulated by the rate-limiting DHODH (Walter and Herr, 2022). DHODH inhibitors are used in the treatment of autoimmune diseases and are currently under investigation for their antiviral and anticancer potencies. In general, DHODH inhibitors prevent cellular proliferation, but some of them, such as BAY 2402234, induce differentiation of acute myeloid leukemia (AML) cells (Christian et al., 2019). BRQ also inhibited tyrosine phosphorylation in lymphocytes which was not corrected by uridine supplementation, suggesting its additional mode of activity (Masuda et al., 2012). Additionally, while the MYC family of oncogenes regulates the expression of DHODH, the key enzymes in *de novo* pyrimidine synthesis, DHODH inhibitors downregulate c-MYC in melanoma, myeloma, and lymphoma cells. Although it is unclear why DHODH inhibition leads to c-MYC downregulation, it is thought to be a result of cell cycle arrest and apoptosis activation (Dorasamy et al., 2017). Or perhaps cellular instability and halted proliferation lead to the deactivation of the MYC family of oncogenes, whose main purpose is to promote proliferation. Another possible scenario is that the inhibition of the DHODH enzyme led to its accumulation or the accumulation of the substrate, which signaled feedback inhibition on the MYC transcription family genes through an unknown mechanism. This makes DHODH inhibitors a potential drug for their anticancer effects in solid and non-solid tumors (Christian et al., 2019). BRQ is the most potent human DHODH inhibitor (IC_50_ = 1.8 nM), with known potential to specifically decrease the proliferation of glioblastoma cells (Xu et al., 1998); and it is in clinical trials for application against COVID-19 and AML (go.drugbank.com/drugs/DB03523).

The tumorigenic potential of iMSCs has been well assessed in previous research (Harada et al., 2021; Yoshimatsu et al., 2021). These cells are typically induced from iNCCs using established protocols and fluorescent cell sorting techniques. While there is currently no evidence to suggest that hiPSCs remain contaminating iMSCs after induction, it is important to demonstrate that hiPSCs can be effectively and selectively eliminated without affecting the characteristics of iMSCs. BRQ has been identified as a chemical that may be able to achieve this goal, and we investigated its potential as a means of purifying iMSCs derived from hiPSCs. We suggest using BRQ as an additional precautionary measure to eliminate unwanted undifferentiated hiPSCs.

In conclusion, it is evident that cell proliferation can be terminated by DHODH inhibition using BRQ. Although we only tested iMSCs reactivity to DHODH inhibition, most terminally differentiated cells could fully utilize the pyrimidine salvage pathway. It has been previously reported that mouse neural stem cells and astrocyte survival are not affected by BRQ (Kondo, 2021). Further investigation of other differentiated cell types is required before this purification method can be fully adopted for hiPSC-derived regenerative cell therapy.

## Data Availability

The datasets presented in this study can be found in online repositories. The names of the repository/repositories and accession number(s) can be found in the article/Supplementary Material.

## Supplementary material

SUPPLEMENTARY FIGURE S1 Unaltered gene expressions of BRQ treated iMSCs **(A)** iMSCs treated for 7 days with BRQ or DMSO, hieratical clustering of samples of the significantly differentially expressed genes (100 nM BRQ vs. DMSO control), bright yellow color indicates higher expression of gene and dark blue for lower expression. **(B)** RT-qPCR of pathway markers for the 7 days BRQ-treated iMSCs.
