# Supplementary figures and images for: Selective vulnerability of human-induced pluripotent stem cells to dihydroorotate dehydrogenase inhibition during mesenchymal stem/stromal cell purification
Source: Front Cell Dev Biol. 2023 Feb 6;11:1089945. doi: 10.3389/fcell.2023.1089945 (PMC9939518; doi:10.3389/fcell.2023.1089945)

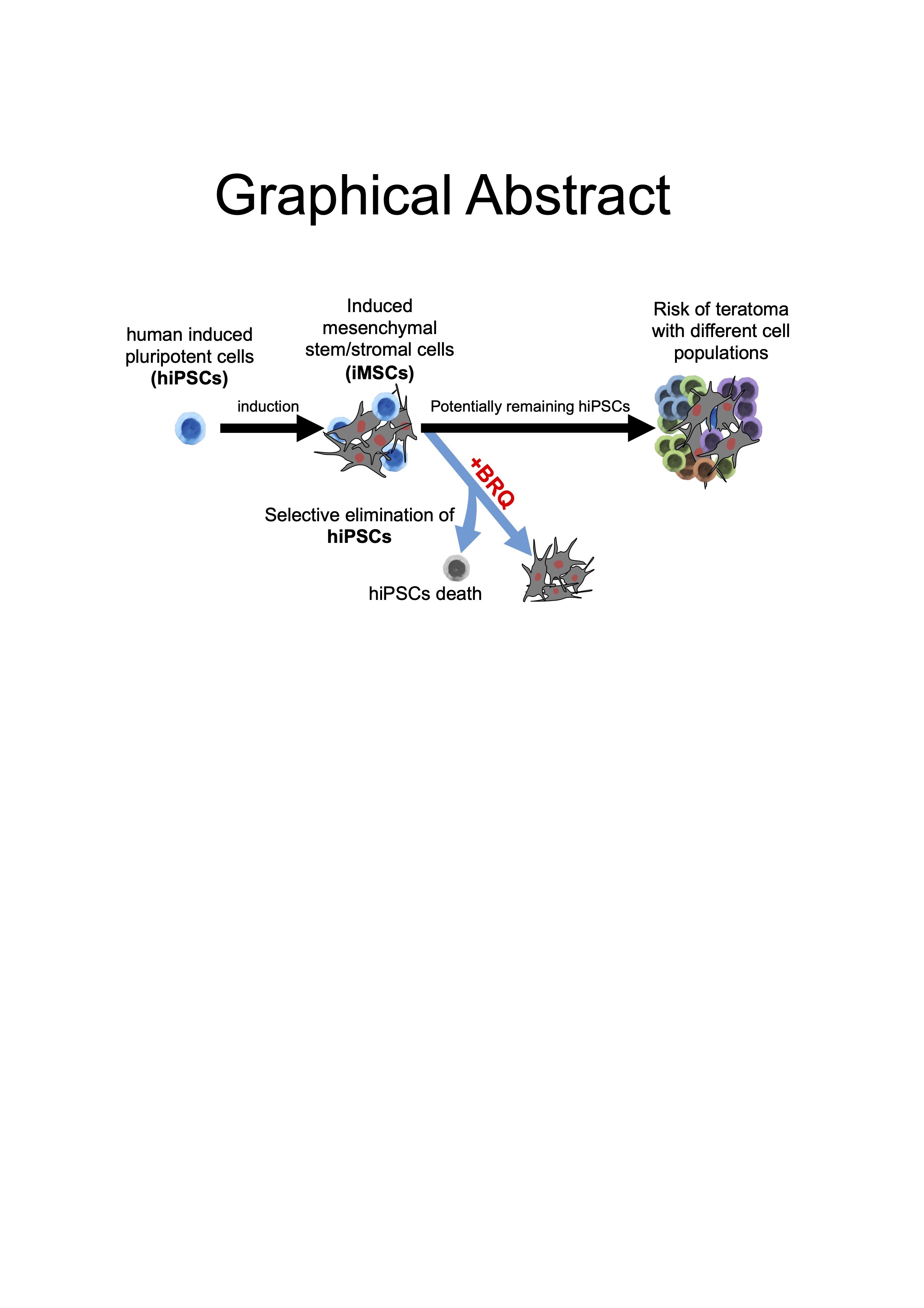

Supplement: Supplementary file 1 [file Image2.jpg]

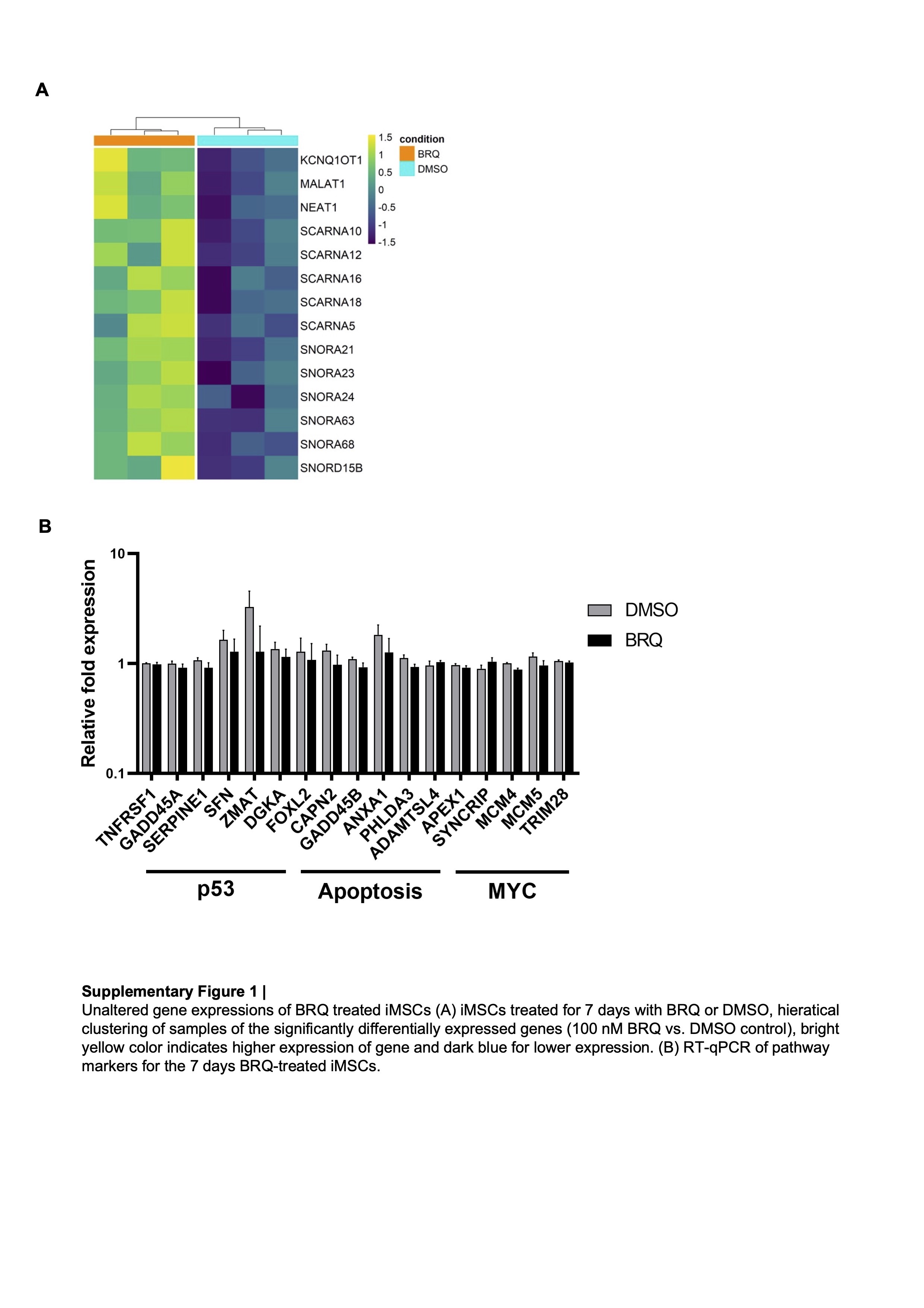

Supplement: Supplementary file 3 [file Image1.jpg]
