# Supplementary material for: Selective vulnerability of human-induced pluripotent stem cells to dihydroorotate dehydrogenase inhibition during mesenchymal stem/stromal cell purification
Source: Front Cell Dev Biol. 2023 Feb 6;11:1089945. doi: 10.3389/fcell.2023.1089945 (PMC9939518; doi:10.3389/fcell.2023.1089945)
Supplement: Supplementary file 2 [file Table1.docx]

Supplementary Material

| Genes | Forward | Reverse |
| --- | --- | --- |
| ACTB | AGGTCTTTGCGGATGTCCACGT | CACCATTGGCAATGAGCGGTTC |
| ADAMTSL4 | CAGAAGGGTTAACGGGCCAC | CGACCCTGTCCTCCAAGTTC |
| ANXA1 | AATCCATCCTCGGATGTCGC | TCAGTGTTTCATCCAGGGGC |
| APEX1 | GCAGATACGGGGTTGCTCTT | TTCGGCATTCCCGTTACGAA |
| CAPN2 | AGTCGCTCTCAAAGGACACG | ACCATGTTTCCCAGTTGGCT |
| CD105 | CACTAGCCCAGGTCTCGAAGG | CTGAGGACCAGAAGCACCTC |
| CD44 | GCAATGCTTCTCAGACCACA | GAGGGGAGAGGGTAGACAGG |
| CD73 | GCCGCTTTAGAGAATGCAAC | CTCGACACTTGGTGCAAAGA |
| CD90 | TGGATTAAGGATGAGGCCCG | CCTAAGTCACTCGCCATCCC |
| DGKA | GAAGAGGTCCAAGCAACGGA | TTGCAGCTGGGCAAAATCAC |
| FOXA2 | TGTTCGAGAACGGCTGCTAC | CCCCGAGTTGAGCCTGTGAG |
| FOXL2 | GCGAAGTTCCCGTTCTACGA | CTCGTTGAGGCTGAGGTTGT |
| GADD45A | TTTTGCCGGGAAAGTCGCTA | AGATGCCATCACCGTTCAGG |
| GADD45B | CCTGCAAATCCACTTCACGC | GTGTGAGGGTTCGTGACCAG |
| IDO1 | GCCAGCTTCGAGAAAGAGTTG | ATCCCAGAACTAGACGTGCAA |
| MCM4 | CTCCGAGCACTATGTCGTCC | GTGGAATCCTCGCCTCTACG |
| MCM5 | ATCGAGAAGCAGCTCAAGCG | ACTTGAGGCGGTAGAGAACC |
| PAX6 | CAGGAAGGAGGGGGAGAGAA | CTTTGCAGCTTCCGCTTCAG |
| PD-L1 | TGGCATTTGCTGAACGCATTT | TGCAGCCAGGTCTAATTGTTTT |
| PHLDA3 | CCCTCGTGTCCTAAACCACC | AGCCAGAGGGAACAACGAAG |
| POU5F1 | GCAAAGCAGAAACCCTCGTG | GATCTGCTGCAGTGTGGGT |
| SERPINE1 | AGAGCGCTGTCAAGAAGACC | AGTTCTCAGAGGTGCCTTGC |
| SFN | CCACTACGAGATCGCCAACA | CGTCCACAGTGTCAGGTTGT |
| SMA | TCCGGAGCGCAAATACTCTG | CCCGGCTTCATCGTATTCCT |
| SYNCRIP | GCGGTAGAGCCGGTTATTCA | TTTGTTGTTGGGCACCTCCT |
| TNFRSF10B | CTGCGCCCACAAAATACACC | GTTCTGTCCCCGTTGTTCCA |
| TRIM28 | GAGGAAGAGGGACGGATTGC | CAGTGGGGTATGCGAACCTT |
| ZMAT3 | TATCGAAGGGAGGGGAGCAA | GGAGGGACTGGAACAACTGG |

**Supplementary Table 1** | Table of forward and reverse primers used to detect the gene expression in alphabetical order.

Figure 1 | (A) iMSCs treated for 7 days with BRQ or DMSO, hieratical clustering of samples of the significantly differentially expressed genes (100nM BRQ vs. DMSO control), bright yellow color indicate higher expression of gene and blue for lower expression. (B) table of forward and reverse primers used to detect the gene expression in alphabetical order.
